# Insights into bovine endometritis with special reference to phytotherapy

**DOI:** 10.14202/vetworld.2017.1529-1532

**Published:** 2017-12-28

**Authors:** Ritika Mandhwani, Anavil Bhardwaz, Sudarshan Kumar, Madhu Shivhare, Ranjit Aich

**Affiliations:** 1 Department of Veterinary Gynecology & Obstetrics, College of Veterinary Science, Mhow, Nanaji Deshmukh Veterinary Science University, Jabalpur-453446, Madhya Pradesh, India; 2 Department of Veterinary Biochemistry, College of Veterinary Science, Mhow, Nanaji Deshmukh Veterinary Science University, Jabalpur - 453446, Madhya Pradesh, India

**Keywords:** ashwagandha, bovine, endometritis, garlic, neem, phytotherapy, tulsi, turmeric

## Abstract

Postpartum reproductive disorders cause heavy economic losses in dairy sector. Uterine infections include endometritis, metritis, mucometra, and pyometra. Postpartum endometritis in dairy cows has been defined as inflammation of endometrium occurring 21 days or more after parturition without systemic signs of illness. The treatment of endometritis with antimicrobials has met with varying degrees of success, inconsistent recovery rate, high cost of treatment, milk disposal, emergence of microbial resistance, and reduced phagocytic activity of polymorphonuclear leukocytes In our country, around 20,000 medicinal plant species have been recorded, but more than 500 traditional communities use about 800 plant species for curing different diseases. Many herbs such as garlic, neem, ashwagandha, and turmeric have been tried for the treatment of endometritis in cows with a good success.

## Introduction

Postpartum reproductive disorders cause heavy economic losses in dairy sector. Uterine infections include endometritis, metritis, mucometra, and pyometra. Among all these uterine inflammatory diseases, endometritis is one of the major gynecological problems affecting reproductive efficacy and economy of milk production in dairy animals. Postpartum endometritis in dairy cows has been defined as inflammation of endometrium occurring 21 days or more after parturition without systemic signs of illness [1]. Clinically, it is characterized by the presence of pus flakes in the uterine discharge. The incidences of clinical and subclinical endometritis in crossbred cows have been reported to be 12% and 29.69%, respectively [2].

The treatment of endometritis with antimicrobials has met with varying degrees of success, inconsistent recovery rate, milk disposal, emergence of microbial resistance, and reduced phagocytic activity of polymorphonuclear leukocytes (PMN cells). The indiscriminate and prolonged use of antimicrobials in the absence of *in vitro* sensitivity tests has contributed to the emergence of resistant strains of bacteria [3].

Use of herbal medicine is becoming popular due to toxicity and side effects of allopathic medicine; hence, clinicians felt an urgent need to find out an alternative therapy for the treatment of uterine infections using immunomodulators and phytotherapeutic measures as a means of activation of natural defense mechanism in the uterus.

Certain plant products have been used as therapeutic agents and have recently become a subject of scientific investigations. The role of many medicinal herbs has been recognized to manage the infertility problem in the past few years. Many herbs such as garlic, neem, ashwagandha, turmeric, and tulsi have been tried for the treatment of endometritis in cows with a good success [4].

In our country, around 20,000 medicinal plant species have been recorded, but more than 500 traditional communities use about 800 plant species for curing different diseases [5].

## Etiology and Pathogenesis

Uterine inflammation is a serious problem in breeding dairy cows worldwide causing considerable economic losses [6]. It is caused by microorganisms multiplying in the endometrium of the uterus. Opportunistic and pathogenic bacteria, that is, *Escherichia coli*, induce mainly cellular local immune response, manifested by leukocyte infiltration visible in a cytological examination. Opportunistic microorganisms in the uterus can secrete substances weakening local immune mechanisms in the endometrium allowing the inflammation to persist for a long time [7].

The bacterial agents commonly isolated from the uterus of postpartum cows are *E. coli, Streptococci* spp.*, Trueperella pyogenes, Bacillus licheniformis, Prevotella* spp., and *Fusobacterium necrophorum*. Studies to evaluate the appearance and odor of vaginal mucus have shown that *T. pyogenes, Proteus* spp., and *F. necrophorum* are associated with purulent or mucopurulent discharge evident in the vaginal mucus while *T. pyogenes*, *E. coli*, and non-hemolytic *Streptococci* are associated with foul-smelling exudates [8].

Impaired immunity of the uterus is considered to be of a key problem in the development of postpartum uterine inflammation, which often persists for 60 days after parturition, negatively affecting reproductive parameters in cowherds [9].

The diameter of the uterine horns indicates the dynamics of uterine involution [10,11].

## Diagnosis

Diagnosis can be done by transrectalpalpation, vaginoscopy, ultrasonography, and endometrial biopsy, but the two most widely used methods are described.

## White side test

In this test, cervical mucus is collected aseptically from the suspected animals and is boiled with an equal amount of 5% sodium hydroxide. The test is considered positive if the color turns into yellow. The intensity of color reaction depends on the number of leukocytes present in the uterine discharge.

Kumar [12] reported that the white side test had a significant positive correlation with pH, bacterial load in cervicovaginal mucus (CVM), and bacterial load in uterine flushing.

### Uterine/endometrial cytology

Two methods are commonly used for uterine cytology: low-volume uterine lavage and cytobrush technique. Both the techniques are based on the percentage of neutrophils in samples obtained.

Ghasemi *et al*. [13] in their study of thirty postpartum cows (28 to 41 days in milk) without signs of clinical endometritis concluded that the endometrial cytobrush technique was successfully used to obtain material for both cytology and RNA extraction, and il8 gene expression may be useful to predict endometrial inflammation.

Honparkhe *et al*. [14] diagnosed subclinical endometritis in buffaloes on the basis of percentage of PMN cells (i.e., if ≥5%) in uterine cytobrush samples which was later confirmed by microbial assay and they concluded that the technique is an efficient and early diagnostic method for subclinical endometritis.

## Natural Immunity: Uterine Defense Mechanism

Effective defense against reproductive tract invasion by environmental organisms is mediated by anatomical and functional barriers as well as nonspecific and specific immune responses [15]. The uterine defense mechanisms against contaminant microorganisms are maintained in several ways: anatomically, by the simple or pseudostratified columnar epithelium covering the endometrium; chemically, by mucus secretions from the endometrial glands; immunologically, through the action of polymorphonuclear inflammatory cells and humoral antibodies.

It is generally accepted that the cyclical pattern of steroid hormone concentration, characteristic for different stages of the estrous cycle, regulates the potential pathogenicity of microorganisms that contaminate the uterus postpartum. For example, the endometrium is more susceptible to infection under progesterone than estrogen dominance. Cattle are resistant to uterine infections when progesterone concentrations are basal and they are susceptible when progesterone concentrations are increased [16].

## Current Therapeutic Measures

### Immunomodulators

In the present scenario, parental and intrauterine treatments with various antibiotics give inconsistent results. Immunomodulators are considered as an alternative approach for treating uterine infections. These substances activate uterine defense mechanism and when infused into uterus initiate local immune system [15]. Some of the different immune modulators used in the treatment of endometritis are as follows:

- *E. coli* lipopolysaccharide
- Oyster glycogen
- Bacteria-free filtrate
- Serum, plasma, or hyperimmune serum
- Levamisole
- Leukotriene B_4_
- Granulocyte-macrophage colony-stimulating factor
- Human recombinant interleukin-8.

### Phytotherapy

The use of herbal remedies for the treatment of livestock dates back to 5000 BC as depicted in *Nakula Samhita*, but this traditional system of medication has picked up importance in the recent years.

#### Garlic: Allium sativum

Historically, garlic has been used for centuries worldwide by various societies to combat infectious disease. Garlic can be provided in the form of capsules and powders as dietary supplements and thus differs from conventional foods or food ingredients. Louis Pasteur was the first to describe the antibacterial effect of onion and garlic juices. One of the active principles of freshly cut garlic homogenates is allicin, which has a variety of antimicrobial activities, and the antibiotic activity of 1mg of allicin is equated to that of 15 IU of penicillin [17].

Sarkar *et al*. [18] evaluated the efficacy of garlic extract and prostaglandin F2 α in the treatment of endometritis in cows. After treatment, there was a significant reduction in bacterial load whereas it was increased in control group. The estrual CVM turned clear in 70% animals treated with garlic extract. The overall conception rate was 50% in treated groups as compared to nil pregnancy in the control group.

Rahi [19] found 50% conception rate and 75% recovery rate of endometritis when treated with aqueous extract of garlic.

#### Neem: Azadirachta indica

Neem is the most commonly used traditional medicinal plant in India. Almost all parts of the neem plant are endowed with medicinal properties, and it has been demonstrated to exhibit immunomodulatory, anti-inflammatory, antifungal, antibacterial, antiviral, and antioxidant properties. Azadirachtin, found only in *Azadirachta* sp., is a complex tetranortriterpenoid. Of all of the liminoids in neem, azadirachtin and its 25 natural analogs are the most biologically active [20].

Kumar *et al*. [21] reported that the hydroalcoholic and hydroacetonic extracts of the neem have potent immune-modulatory and therapeutic efficacy on endometritis in repeat breeding crossbred cows. In their study, a significant rise was found in total leukocyte count (TLC), PMN, and immunoglobulin concentration in both the treated groups.

#### Tulsi: Ocimum sanctum

*O. sanctum* L., known as “tulsi” in Hindi and “holy basil” in English, is an erect softy hairy aromatic herb or undershrub found throughout India. *O. sanctum* L. is held sacred by Hindus and is used as a medicinal plant in day-to-day practice for various ailments. In Ayurveda, tulsi has been well documented for its therapeutic potentials. Different parts of tulsi plant, for example, leaves, flowers, stem, root, and seeds, are known to possess therapeutic potentials and have been used by traditional medical practitioners as expectorant, analgesic, anticancer, antiasthmatic, antiemetic, diaphoretic, antidiabetic, antifertility, hepatoprotective, hypotensive, hypolipidemic, antimicrobial, and antifungal activity against *Aspergillus niger*[22].

Eugenol is a phenolic compound and major constituent of essential oil extracted from different parts of tulsi plant. Eugenol is the most prominent phytoconstituent present in the tulsi plant, which may be responsible for antimicrobial activity [23].

#### Ashwagandha: Withania somnifera

Ashwagandha is an important herb in the ayurvedic and indigenous medicinal system for over 3000 years.

It contains potentially active constituents such as *Sitoindosides VII-X* and *Withaferin A*. It is a sedative, diuretic, anti-inflammatory agent, is generally used for increasing energy and endurance, and acts as an adaptogen that exerts a strong immune-stimulatory and antistress effects [24].

Owis *et al*. [25] found the antibacterial activity of alcoholic extract made by ashwagandha roots against *Salmonella typhimurium*. Moreover, the extract did not induce lysis on incubation with human erythrocytes advocating their safety to the living cells.

#### Turmeric: Curcuma longa

Turmeric is an ancient spice derived from the rhizomes of *C. longa* and is also known as “golden spice of India.” It has been found that both curcumin and the oil fraction suppress the growth of several bacteria such as *Streptococcus, Staphylococcus* and *Lactobacillus* [26].

## Phytotherapy in Combination

Owis *et al*. [25] in their studies showed that ashwagandha and garlic are antibacterial and immune-modulatory in nature and thus reduce bacterial load and subsequently inflammation process.

Kumar [4] found better therapeutic effects of garlic, neem, and garlic+turmeric+neem; this was evident by observing significant increases in TLC and PMN% in uterine flushing after treatment with herbs.

Garlic + ashwagandha extract was found as the most effective treatment among all treated groups and, thus, can replace conventional antibiotic in future for bacterial endometritis leading to repeat breeding condition in crossbred cows [27].

Singh [28] found that the cows exhibited clear discharge with ciprofloxacin (93%) followed by garlic (88.33%) and neem (86.67%) after treatment and found a significant difference in PMN%, TLC, and lymphocyte and monocyte counts.

## Conclusion

Uterine dysfunction can have a major impact on the profitability of a dairy operation. Timely and accurate diagnosis is essential to ensure appropriate management of uterine infections.

Endometritis is one of the major gynecological problems affecting reproductive efficiency and economy of milk production in dairy animals. Due to the emergence and spread of microbial resistance, there exists a need for the development of new antimicrobial of natural origin. Apart from microbial sources, plants such as garlic, tulsi, neem, ashwagandha, and turmeric appear to be valuable antimicrobial resources.

The compelling need to find an alternative therapy using natural resources for the treatment of uterine infections is necessary for the activation of natural defense mechanism in the uterus.

## Authors’ Contributions

RM conceptualized, designed, and wrote the manuscript. SK contributed in literature collection and reviewed the manuscript. SK made critical comments and helped in revising the manuscript. AB, MS, and RA edited and made critical comments on the manuscript. RM and SK conceptualized, designed, made critical comments on the revised manuscript and edited the manuscript for the final version. All authors read and approved the final manuscript.
